# Prevalence of Orthohantavirus-Reactive Antibodies in Humans and Peri-Domestic Rodents in Northern Ethiopia

**DOI:** 10.3390/v13061054

**Published:** 2021-06-02

**Authors:** Yonas Meheretu, Åsa Granberg, Gebregiorgis Berhane, Hussein Khalil, Olivia Wesula Lwande, Mengistu Mitiku, Kiros Welegerima, Joëlle Goüy de Bellocq, Josef Bryja, Hagos Abreha, Herwig Leirs, Frauke Ecke, Magnus Evander

**Affiliations:** 1 Department of Biology, Mekelle University, Mekelle P.O. Box 3102, Ethiopia; gebregergsbr28@gmail.com (G.B.); kiros.welegerima@mu.edu.et (K.W.); 2 Institute of Mountain Research & Development, Mekelle University, Mekelle P.O. Box 231, Ethiopia; 3 Institute of Vertebrate Biology of the Czech Academy of Sciences, 603 65 Brno, Czech Republic; joellegouy@gmail.com (J.G.d.B.); bryja@brno.cas.cz (J.B.); 4 Department of Epidemiology and Global Health, Umeå University, 901 85 Umeå, Sweden; asa.granberg@gmail.com; 5 Department of Wildlife, Fish, and Environmental Studies, Swedish University of Agricultural Sciences, 901 83 Umeå, Sweden; hussein.khalil@slu.se (H.K.); frauke.ecke@slu.se (F.E.); 6 Department of Clinical Microbiology, Virology, Umeå University, 901 85 Umeå, Sweden; olivia.lwande@umu.se (O.W.L.); magnus.evander@umu.se (M.E.); 7 College Health Sciences, Mekelle University, Mekelle P.O. Box 231, Ethiopia; mengistu.mitiku@mu.edu.et (M.M.); hagosteka@gmail.com (H.A.); 8 Department of Zoology and Fisheries, Faculty of Agrobiology, Food and Natural Resources, Czech University of Life Sciences Prague, 165 21 Prague, Czech Republic; 9 Evolutionary Ecology Group, University of Antwerp, 2610 Wilrijk, Belgium; herwig.leirs@uantwerpen.be

**Keywords:** orthohantavirus, rodents, rural community, risk factors, Ethiopia

## Abstract

In 2012, Tigray orthohantavirus was discovered in Ethiopia, but its seasonal infection in small mammals, and whether it poses a risk to humans was unknown. The occurrence of small mammals, rodents and shrews, in human inhabitations in northern Ethiopia is affected by season and presence of stone bunds. We sampled small mammals in two seasons from low- and high-density stone bund fields adjacent to houses and community-protected semi-natural habitats in Atsbi and Hagere Selam, where Tigray orthohantavirus was first discovered. We collected blood samples from both small mammals and residents using filter paper. The presence of orthohantavirus-reactive antibodies in blood was then analyzed using immunofluorescence assay (human samples) and enzyme linked immunosorbent assays (small mammal samples) with Puumala orthohantavirus as antigen. Viral RNA was detected by RT-PCR using small mammal blood samples. Total orthohantavirus prevalence (antibodies or virus RNA) in the small mammals was 3.37%. The positive animals were three *Stenocephalemys albipes* rats (prevalence in this species = 13.04%). The low prevalence made it impossible to determine whether season and stone bunds were associated with orthohantavirus prevalence in the small mammals. In humans, we report the first detection of orthohantavirus-reactive IgG antibodies in Ethiopia (seroprevalence = 5.26%). *S. albipes* lives in close proximity to humans, likely increasing the risk of zoonotic transmission.

## 1. Introduction

Orthohantaviruses (family *Hantaviridae*, genus *Orthohantavirus*) are RNA viruses that cause severe zoonotic diseases in humans, and several new species of ortho-, mobat- and loanhantaviruses have been discovered during the last decades. In Europe and Asia, species of orthohantaviruses cause hemorrhagic fever with renal syndrome (HFRS), while in the Americas, other species cause hantavirus cardiopulmonary syndrome (HCPS) [1]. However, recent studies refute this transatlantic dichotomy between HFRS and HCPS and reveal substantial clinical overlaps between the two diseases [2,3,4]. In Africa, serological evidence for orthohantavirus infections in rodents and humans have since the 1980s been reported in more than a dozen countries see for more [4]. Since the Sangassou virus, carried by the murid African wood mouse (*Hylomyscus simus),* was isolated from Guinea [5], several novel orthohantavirus strains have been isolated or identified in Africa, including Seoul orthohantavirus in wild roof rats (*Rattus rattus*) in Senegal [6]) and Tigray orthohantavirus (TIGV) in white-footed Ethiopian rats in northern Ethiopia [7]. In humans, most recent serological studies have also found antibodies against orthohantaviruses in West Africa (Guinea) [8,9], South Africa [10] and East Africa (Tanzania) [11]. So far, orthohantaviruses have not been confirmed to cause disease in humans in sub-Saharan Africa, but one likely case has been reported in the same area where the Sangassou virus was detected [8].

The majority of orthohantaviruses are carried by rodent reservoir hosts, but several novel viruses belonging to the different genera of the Hantaviridae family have been discovered in soricomorphs (shrews and moles) and bats [12]. Most species and strains of hantaviruses appear to be restricted to one or a few closely related animal species as natural reservoir hosts [13,14,15]. The transmission route for orthohantaviruses to humans starts when the infected hosts shed the virus in saliva, urine and/or feces. Humans are typically infected by inhaling virus-contaminated aerosols of host excreta or via direct contacts such as bites [9,16]. Human-to-human transmission is rare and so far limited to Andes orthohantaviruses (see [17]).

The factors determining zoonotic risk can be classified into three overarching categories [18]. The first category relates to factors that determine the prevalence of the virus in the reservoir population. Such factors could be the dynamics of the reservoir species population, transmission mechanisms among the reservoir populations, assemblages of co-occurring species including predators, behavior and environmental factors such as land-use and climate [19,20]. Land use and climate may not only affect the virus prevalence in the host population, but it might also alter, for instance, the food and/or shelter seeking behavior among the hosts, forcing them into closer contact with humans. These drivers are related to the second category, which considers factors that influence how likely humans are to encounter the virus. For TIGV, such factors could be household and agricultural practices around human inhabitations, whether the reservoir rodent infests people’s houses or not, and how people behave upon encountering a rodent or its excreta [21]. The third category relates to the probability of infection once the virus is encountered. This is determined by factors such as immune defense, presence of receptor cells on host cells, ability (presence) of a host cell to support the viral replication, the viral strain/genotype, and the viral dose and the duration and proximity of contact [18]. Knowledge about the factors important for infection, in combination with prevalence or incidence data, are crucial for mitigating and preventing transmission and infection risk.

TIGV was discovered for the first time in 2012 during a screening of small mammals from domestic and pre-domestic areas in the Tigray province of northern Ethiopia [7]. The virus was found in the endemic white-footed Ethiopian rat (*Stenocephalemys albipes*), a typical montane forest rodent species that also lives in shrublands and crop fields close to human settlements [7,22,23]. TIGV’s complete genome has been characterized, revealing a typical orthohantavirus organization [24]. Recently, Meheretu et al. (2019) reported the occurrence of TIGV in two sister host species (*S. albipes* and *S. zimai*) that likely have evolved by ecological speciation at different elevational zones in the northern part of the Ethiopian Highlands [25,26].

In the same area where TIGV was first discovered, an earlier study investigated the rodent abundance and population dynamics in relation to density of stone bunds (stone walls mainly constructed in fields to curb soil erosion by runoff) which rodents use as shelter and corridor [27]. We found that large-scale stone bund building programs adopted in the Tigray highlands to prevent soil erosion since the 1980s [28] have resulted in a higher abundance of rodents [27]. Thus, living in an area with a high proportion of a favorable rodent habitat, in this case, high stone bund density, might increase the risk of encountering the virus and consequently the risk of infection. However, if TIGV does infect humans, several other additional factors are also likely to influence the human risk of being infected by the virus.

Thus, the aims of this study were (i) to investigate the assemblage of small mammal species occurring around human inhabitations in two rural communities (Atsbi and Hagere Selam) where TIGV was first discovered and in relation to season and density of stone bunds. We also investigated (ii) the prevalence of antibodies against orthohantavirus in small mammals and (iii) humans sampled from the two rural communities, and (iv) examined the association between the seroprevalence and potential risk factors. We hypothesized that small mammal abundance will be higher in the post-rainy season and areas with a high density of stone bunds compared to in the dry season and areas with a low density of stone bunds. Further, we expected that the prevalence of antibodies against orthohantavirus will be significantly higher in both small mammals and humans in areas with a high density of stone bunds compared to areas with a low density of stone bunds.

## 2. Materials and Methods

### 2.1. Study Area

**Figure 1:**
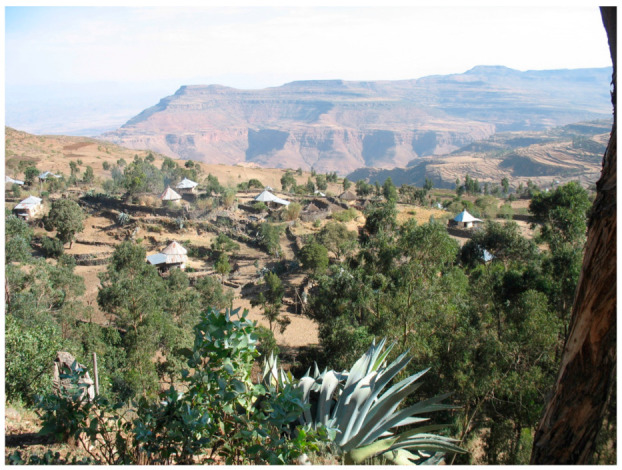
Landscape picture of part of the study area in Hagare Selam. Pointed tin roofs approximately represent one household. Stone bunds are visible between households and, in the back of the picture, in the fields (Photo: Å. Granberg).

**Figure 2:**
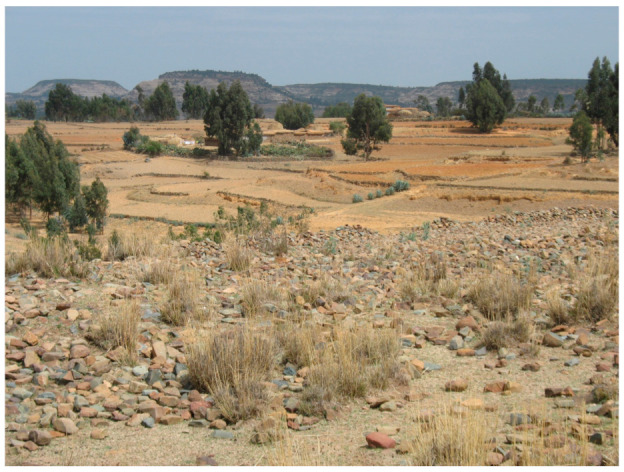
Landscape picture of part of the study area in Atsbi. In the picture two households with flat, straw covered roofs are visible, surrounded by stone bunds that also delineate the fields (Photo: Å. Granberg).

The study was conducted in two rural villages where TIGV was first discovered: Golgolnaele (13°52′51.91″N, 39°43′30.36″E, average elevation 2700 m above seas level (a.s.l.)), ca. 1 km north-east of the rural town of Atsbi, and Mahbere Silassie (13°39′50.97″N, 39°08′16.29″E, 2,600 m a.s.l.), ca. 3 km north-east of the rural town of Hagere Selam, in the Tigray Province, Ethiopia. The land use in both study areas is dominated by crop fields (ca. 60%) in flat areas and rangeland and enclosures in steep slopes (Figure 1 and Figure 2). Enclosures (also called exclosures) are protected communal semi-natural habitats where grazing and farming are prohibited. The remaining native vegetation is largely composed of patchy bushes and scrubs. The residents of both study areas were, as a large part of the Ethiopian population, rural farmers with a livelihood consisting of rainfed, small-scale subsistence agriculture with an average farm size of ca. 1 to 1.5 ha [29]. The main crops grown are wheat (*Triticum aestivum*), barley (*Hordeum vulgare*), tef (*Eragrostis tef*) and different kind of pulses.

In the Atsbi area, the average population density was ca. 74 persons per square km. The mean annual precipitation was 600 mm. The mean annual max temperature was between 20 and 21 °C and min temperature between 8 and 10 °C. In the Hagere Selam area, the average population density was ca. 107 persons per square km. The mean annual precipitation was 700–750 mm. The mean annual max temperature was between 20 and 22 °C and min temperature was between 4 and 6 °C. Both areas represent the highland agroecology, with a tropical monsoon climate with wide topographically induced variations in climatic conditions. The rainfall is uni-modal and erratic, much of it concentrated between June and mid-September, which are the main rainy months.

### 2.2. Small Mammals Sampling

In each study area, three 60 × 60 m square grids were set, one each in low stone bund (LSB) and high stone bund (HSB) density crop fields adjacent to the houses of the farmers and one in an enclosure away from houses. We defined the LSB density grids as those with stone bunds spaced ca. 15 m mean distance, and HSB density grids as those with stone bunds spaced ca. 10 m mean distance (for details, see [27]). In each grid, a combination of 28 Sherman and 21 snap traps were set, each separated by a 10 m interval, for three consecutive days and nights in March 2017 in the dry season and in September 2017 in the post-rainy season. Traps were baited with peanut butter mixed with canned tuna fish, checked for captures twice a day, early in the morning (ca. 07:00 h) and late in the afternoon (ca. 18:00 h). For each capture, grid type, trap type and trap station were recorded. Captures were examined for sex and sexual conditions. Females were considered reproductively active when they exhibited perforated vagina, and/or were pregnant or lactating; and reproductively inactive when vagina was plugged. Males were considered reproductively active when they exhibited scrotal testes and reproductively inactive when they exhibited abdominal testes. Species identification and common names of the small mammals followed [30,31]. Live-trapped animals were euthanized by cervical dislocation in accordance with the ethical policies and guidelines of the Committee for Animal Care and Use (Mekelle University, Ethiopia) and blood was taken from the heart in specially designed filter paper (Nobuto Blood Sampling Paper, Toyo Roshi Kaisha Ltd., Tokyo, Japan). Small mammal abundance (number of individuals captured) was recorded per study area, grid type and season. We refrained from performing further estimates, such as density, trap success and species diversity, to avoid bias because of low capture rates and seroprevalence.

### 2.3. Human Blood Sampling and Questionnaire

In a cross-sectional study, we collected blood samples from humans for antibody analysis and applied short open-ended questionnaires for risk assessment in March 2017. The potential participants in the serosurvey from both study areas were randomly contacted by visits to their homes, in a house-to-house approach. Blood samples were collected from eligible participants by puncturing their finger using a disposable mini lancet by soaking the specially designed filter paper with flowing blood. To be eligible for participation, residents had to be ≥18 years old and living in the study areas. For each household, a GPS (global positioning system) location was recorded to be able to assess the surrounding density of the stone bunds.

### 2.4. Assessment of Variability in Stone Bund Density

To link the presence of orthohantavirus-reactive antibodies in the humans to favorable rodent habitat, stone bund density (SBD) was estimated in a GIS (geographic information system) using QGIS within a 100 m radius around each participant’s households. The digitalization of stone bunds was done based on visual identification in QGIS (version 2.12.0-Lyon) using free background satellite images via Open Layers Plugin from Google Maps (Google Satellite, Bilder © 2021, CNES/Astrium DigitalGlobe) and Bing (Bing Aerial, © 2021 DigitalGlobe, © 2021 GeoEye, © 2021 Microsoft Corporation). Before digitalization, calibration was done in the field by comparing visibility of stone bunds in the satellite images with what could be seen in the field. Stone bunds were rows (linear objects) of stacked stones, at least two stones high, which could be identified on the satellite images.

Buffers around each household were generated in a GIS by first transforming the gpx-files with the waypoints from the GPS to a shapefile with coordinate system Adindan/UTM zone 37N (EPSG:20137), which is a suitable projected coordinate system for the study area. Buffers with 100 m radius were then generated using the vector-based geoprocessing tool in QGIS with the waypoint shapefile as centroid data. By combining the digitalized data of stone bunds with the buffers, the total stone bund length per individual buffer could be calculated. This was done using the vector-based analytic tool in QGIS.

Stone bund density was possible to quantify from the satellite images. To get more detailed data on the suitability of stone bunds from a rodent perspective, we additionally quantified the width, length and density of the stone bunds using high resolution remote sensing data. For this, we surveyed the study areas with a drone (DJI Mavic 2 Pro) on 21 September 2019. As a ground station and for planning the remote sensing missions, we used the Pix4Dcapture software (pix4D.com). Flying altitude was 50 m, the along- and across-track image overlap was set to 80%, and the missions were performed as a double grid. To create orthomosaics and digital elevation models (DEM), we processed the generated images in AgiSoft Metashape Professional© (version 1.5.5). The spatial resolution of the generated orthomosaics was set to 4 cm and that of the DEM to 3 cm. Visible stone bunds were digitized in ArcGIS (version 10.6; ESRI Inc., Redlands, CA, USA) from the orthomosiacs at a scale of 1:100 within a 100 m buffer surrounding the villages. To distinguish paths from stone bunds in the orthomosaics, we used the DEM, since paths were visible as depressions in the DEMs, while stone bunds were visible as increments.

### 2.5. Viral Screening

#### 2.5.1. Detection of Human IgG Antibodies

Human blood was eluted from the filter papers in 1 mL phosphate buffered saline (PBS) pH 7.4 overnight at 20 °C. We analyzed the presence of orthohantavirus-reactive antibodies in the eluted blood samples with an indirect immunofluorescence assay (IFA) used in routine diagnostics at Norrland’s University Hospital, Umeå, Sweden. PUUV orthohantavirus (PUUV) cultured in Vero cells was used as an antigen, thus detecting antibodies directed against both the PUUV nucleocapsid protein and glycoproteins. We speculated that antibodies against PUUV would cross-react with other types of orthohantaviruses, and previously other known orthohantaviruses have been used as antigens for detecting antibodies for new orthohantaviruses, e.g., [10,32]. Furthermore, PUUV infected cells could be prepared in BSL-2 conditions (classified as a BSL-2 agent in Sweden). Other orthohantaviruses (e.g., Seoul-, Hantaan-, Dobrava-Belgrade orthohantavirus) would need BSL-3 conditions. Neither TIGV nor any TIGV-specific proteins were available. Thus, we did not make use of other orthohantaviruses [24]. Details of the IFA assay used to detect orthohantavirus-reactive antibodies in the human blood samples is described in [33].

#### 2.5.2. Detection of Rodent IgG Antibodies

Small mammal blood was eluted from the filter papers in 1 mL PBS overnight at 20 °C. Presence of orthohantavirus-reactive antibodies in the blood eluate samples were analyzed by enzyme-linked immunosorbent assay (ELISA). To detect orthohantavirus-reactive IgG antibodies, the PUUV nucleocapsid protein was used as antigen, as described in [32].

#### 2.5.3. Viral RNA Analyses

Total viral RNA was extracted from the dry blood spots of the small mammals using QIAamp viral RNA Mini Kit (QIAGEN). The RNA samples were screened for orthohantavirus RNA as in [15], targeting a 347 nucleotide-long part of the polymerase (L) gene of the orthohantavirus genomes. First, primers Han-L-F1: 5′-ATGTAYGTBAGTGCWGATGC-3′ and Han-L-R1: 5′-AACCADTCWGTYCCRTCATC-3′ were used for a one-step RT-PCR before performing a nested-PCR using primers Han-L-F2: 5′-TGCWGATGCHACIAARTGGTC-3′ and Han-L-R2: 5′-GCRTCRTCWGARTGRTGDGCAA-3′ [5]. Only one positive sample was obtained, and the PCR product was Sanger sequenced with the forward primers Han-L-F2. The sequence chromatogram was visually inspected in Geneious 8.1.9 and compared to Tigray sequences from previous studies [7,15].

#### 2.5.4. Risk Factors for Human Infection

Nine potential risk factors were considered as independent variables and were tested for their association with the response variable, viz. serostatus (Table 1). For the association between serostatus and potential risk factors, see also [34,35]; study site, gender, age, cat ownership, contact with rodents and rodent bite were tested separately with bivariate logistic regression. The outcomes from the logistic regression were presented as odds ratios (OR) together with the corresponding 95% confidence intervals (CI) and the *p*-value of the association as a measure of statistical significance. The association between being seropositive and the variable sightings of rodents could not be tested with logistic regression because one of the categories lacked seropositive samples. This association was instead tested separately with Fisher’s exact test and the outcome was presented with the *p*-value. A two-sample t-test was used to test whether average stone bund density around the human dwellings differed between seropositive and seronegative participants and the outcome was presented with t-value and *p*-value.

Because of the low number of positive blood samples, it was not possible to make adjustments for potential covariation in the statistical tests. However, associations between the independent variables could be expected and knowledge about the strength of such associations could also be of importance both for a potential expansion of this study and for interpretation of results from the logistic regression. Therefore, the potential associations between the independent variables were tested with chi-square test or Fisher’s exact test in cases where both variables were categorical and with t-test in cases where one of the variables was quantitative. Results were statistically significant if *p* ≤ 0.05. Statistical analyses were carried out using STATA, ver. 14.

## 3. Results

### 3.1. Composition of Small Mammals

We captured a total of 104 small mammals, 56 in Atsbi and 48 in Hagere Selam. The small mammals belonged to seven rodent and one shrew species (Table 2). In Atsbi (characterized by higher densities of total and medium-sized stone bunds outside the village and longer length of overall and big-sized stone bunds in the village and medium- and big-sized stone bunds outside the village), the African grass rat (*Arvicanthis niloticus,* formerly *A. dembeensis* [25]) was the most abundant species (*n =* 25; 44.6%). In contrast, in Hagere Selam (characterized by higher densities of total and big-sized stone bunds in the village and higher density and longer length of stone bunds outside the village), the white-footed Ethiopian rat (*Stenocephalemys albipes*) was the most abundant species (*n =* 14; 29.2%). We captured nine individuals of the white-footed Ethiopian rat in Atsbi. While more (*n =* 18) small mammals were captured in the high stone bund density fields than in the low stone bund density fields (*n =* 10) in Atsbi (Table 2), an almost equal number of small mammals were captured in the high and low stone bund density fields (*n =* 19 and *n =* 20, respectively) in Hagere Selam. While we captured more small mammals in the dry season (*n =* 36) than in the post-rainy season (*n =* 20) in Atsbi, there was an equal number of small mammals (*n =* 24) captured in the dry and post-rainy seasons in Hagere Selam (Table 3).

### 3.2. Prevalence of TIGV in Small Mammals

Of the 104 small mammals, 89 rodent dry blood samples could be analyzed for seroprevalence using the ELISA (51 from Atsbi and 38 from Hagere Selam), and two individuals of *Stenocephalemys albipes* were found to contain antibodies that reacted with PUUV, resulting in a seroprevalence of 2.25% in the total study population. In Hagere Selam (where more white-footed Ethiopian rats were captured), the seroprevalence was 5.26% compared to 0% in Atsbi. Considering seroprevalence only for the white-footed Ethiopian rat, one of the two known hosts of TIGV to date [15], seroprevalence was 8.69% (2/23) in the total white-footed Ethiopian rat population and 14.28% (2/14) for the same species captured only in Hagere Selam. The positive individuals were a lactating adult female (body mass 92 g) and a sub-adult female (body mass 29 g) captured in the post-rainy and dry seasons, respectively, in the same HSBD field in Hagere Selam. The field was covered with wheat crop during the post-rainy season but left fallow during the dry season.

The RT-PCR analysis of the 89 samples resulted in one positive sample, in addition to the two antibody-positive samples. The obtained sequence showed only one synonymous mutation compared to Tigray orthohantavirus strain 97 (GenBank AN: JQ956486) found in *Stenocephalemys albipes* in Hagere Selam nine years before [7]. The new sequence was deposited in GenBank (AN: MK875671). The positive individual was *S. albipes*, an active adult male (body mass 97 g) captured in the post-rainy season in the enclosure (acacia bushland) in Hagere Selam.

### 3.3. Seroprevalence in Humans

In total, 119 people from 76 households agreed to participate in the study. Of these, blood samples from 114 people could be analyzed for PUUV reactive antibodies. Of the 114 samples analyzed, 49 came from Atsbi and 65 came from Hagere Selam (Table 4). In both study areas, more women than men participated, with a total distribution of 78 women and 36 men among analyzed samples. The participants were between 18 and 80 years old, with most participants in the age group of 18–40 and fewest in the age group 61–80, and participating men were on average significantly older than women (t = −2.2, *p* = 0.03). Neither the sex distribution (χ^2^ = 0.037, df = 2, *p* = 0.8) nor the age distribution (χ^2^ = 3.329, df = 2, *p* = 0.19) differed between the two study areas. Among the 114 samples, six were found to contain PUUV reactive antibodies, giving a seroprevalence of 5.26% in the total study population. In Atsbi the seroprevalence was 10.20% (5/49) compared to 1.54% (1/65) in Hagere Selam (Table 4).

### 3.4. Risk Factors

**Figure 3:**
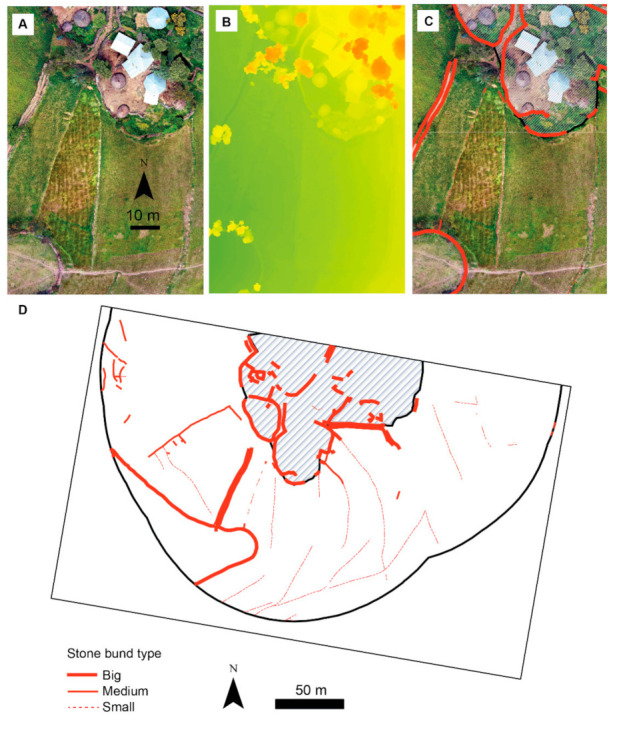
Illustration of stone bund identification in a high resolution (4 cm) orthophotograph of the study area in Hagere Selam. (**A**) Section of the orthophotograph showing the boundaries between the village and the surrounding arable fields including stone bunds. (**B**) Digital elevation model (DEM) with a spatial resolution of 3 cm and altitude of objects ranging between 2438 and 2489 m. (**C**) Stone bund (red lines) digitized in and outside the village. (**A**–**C**) show the same section of the study area. (**D**) Stone bunds digitized for the whole study area in Hagere Selam. The shaded area represents the village, and the black lines delineated the 100 m buffer area within which stone bunds outside the village were digitized. The width of the big-sized stone bunds is >40 cm, that of medium-sized ones is 20–40 cm and small-sized stone bunds have a width <20 cm and generally have only one or two layers of stones.

Because of the small sample size and low seroprevalence, the statistical power was low when testing for associations between seroprevalence and human risk factors. None of the associations was statistically significant but there was a trend for seropositivity being more likely when a household was surrounded by young stone bunds (Table 4). Stone bund density (SBD) differed between the two study areas, with a significantly higher SBD in the village in Hagere Selam than in Atsbi (*p* = 0.000, Table 5 and Table 6). Therefore, the association between being seropositive and SBD were tested separately for each of the study areas. The separate tests showed diametric results; in Atsbi, people that were seropositive on average lived in houses with LSBD in contrast to those that were seronegative. In Hagere Selam, on the other hand, the only person that was seropositive had HSBD around the house which was higher than the SBD around the houses of seronegative individuals. The majority of stone bunds were built more than 10 years ago, but the risk of being seropositive was higher among those who reported that their stone bunds were built less than 10 years ago (see trend in Table 4). Stone bund density was lower around households with young stone bunds (t = −1.4, *p* = 0.08). However, the reported age of the stone bunds did not differ significantly between the study areas (χ^2^ = 1.005, df = 1, *p* = 0.3). Density of stone bunds (DSB) outside the village was higher in Atsbi than in Hagere Selam (ca. 437 m/ha and 396 m/ha, respectively); however, inside the village, DSB was higher in Hagere Selam than in Atsbi (ca. 134 m/ha and 91 m/ha, respectively), which resulted in almost no difference in total DSB (−5.5 m/ha) (Figure 3, Table 6). However, outside the village, Atsbi was characterized by higher density of medium-sized stone bunds (ca. 206 m/ha), but lower density of the small-sized stone bunds (ca. 63 m/ha). In the village, the density of the big-sized stone bunds was larger in Hagere Selam (ca. 132 m/ha) than in Atsbi (83 m/ha).

The total length of stone bunds was longer in Atsbi (ca. 4875 m) than in Hagere Selam (ca. 2070) (Figure 3, Table 6). Outside the village, Hagere Selam was characterized by longer length of the small-sized stone bunds, but shorter lengths of the medium and big-sized stone bunds. In the village, the length of the big-sized stone bunds was longer in Atsbi than in Hagere Selam.

The presence of domestic cats in the households differed between the two study areas: only 3 out of 49 (6%) of the participants in Atsbi were living without a cat in the household, while in Hagere Selam 42 out of 65 (65%) were living without a cat (Fisher’s exact test: *p* = 0.000). However, the odds of being seropositive were not significantly different between those who did not have a cat and those with a cat (Table 4). All participants reported that they have seen rodents, but sighting locations varied and included domestic, peri-domestic and grain storage areas. While not significant, all seropositive samples came from people who reported that they saw rodents in their house or in grain storage areas while none of the people that reported only seeing rodents in the field or elsewhere were seropositive (Table 7). Significantly more people in Hagere Selam reported that they never saw rodents at home or in the grain storage (χ^2^ = 7.75, df = 2, *p* = 0.005). Furthermore, women reported more often than men that they never saw rodents in their home or storage (Fisher’s exact test: *p* = 0.000). Seeing rodents in their own house or storage was also associated with age: people in the youngest and oldest age groups more frequently reported never seeing rodents in the house or storage area than people in the middle age group (Fisher’s exact test: *p* = 0.06). Whether a person ever saw rodents in or around their house or grain storage area was not associated with having a cat.

People who reported seeing rodents in or around houses or grain storage areas have also had contact with rodents more frequently (Fisher’s exact test: *p* = 0.02) and men have had contact with rodents more often than women (x^2^ = 7.09, df = 3, *p* = 0.03), but there was no difference between men and women in being bitten by rodents. More people had been bitten by rodents in Atsbi (Fisher’s exact test: *p* = 0.009) and having been bitten was also proximate to being significantly associated to stone bunds ≤ 10 years old (Fishers exact test: *p* = 0.06).

## 4. Discussion

### 4.1. Small Mammal Abundance and Diversity

A total of seven species of rodents and one species of shrew were captured in both study areas during the study period. Previous studies in the same study areas reported the same species assemblages [7,22]. As expected, more small mammals were captured in the HSBD grid (*n =* 18) than in the LSBD grid (*n =* 10) in Atsbi. This result supports the general hypothesis that fields with HSBD likely provide better cover against potential predators than fields with LSBD, hence harboring more rodents [27]. However, in Hagere Selam, there was no difference in rodent abundance between HSBD and LSBD grids. We suspect this is partly because of the interference of residents with the traps set at HSBD grid during the night, which contributed to loss of traps and possibly to the low captures.

The African grass rat was the most abundant species in Atsbi (*n =* 25; 44.6%). The species requires a significant cover for its diurnal living habit [36]. Previously, significantly more individuals of this species have been trapped in crop fields with HSBD than fields with LSBD [27]. It regularly co-occurs with the white-footed Ethiopian rat and the Awash multimammate mouse (*Mastomys awashensis*) in these habitats [23,27]. Nonetheless, in Hagere Selam, the white-footed Ethiopian rat, the reservoir for TIGV, was the most abundant species (*n =* 14; 29.2%). In the highlands of northern Ethiopia, the species regularly occurs in a variety of habitats including montane forests, bushlands, agricultural fields, peri-domestic and domestic habitats [22,23,27].

Numerically more small mammals (*n =* 36) were captured in the dry season than in the post-rainy season (*n =* 20) in Atsbi and all shrew individuals (*n =* 9) were captured only during the post-rainy season when the fields were covered with crops. The higher number of small mammals captured in the dry season than in the post-rainy season in Atsbi contradicts an earlier report [27]. Generally, in Ethiopian highlands, rodent abundance peaks more during the post-rainy season than the dry season during years of good rains. We suspect the unusual drop in small mammal captures in the post-rainy season was linked to the unusually poor rainfall the study areas experienced during the previous wet (rainy) months (June to August).

### 4.2. Tigray Orthohantavirus: Seroprevalence and Risk Factors

The overall prevalence of TIGV was lower than the 17.85% (10/56) TIGV prevalence reported previously for the white-footed Ethiopian rat from peri-domestic habitats in the same study areas with a molecular screening [7]. The study confirmed the occurrence of TIGV in the white-footed Ethiopian rat, here in crop fields and acacia bushland in both dry and post-rainy seasons. Given that particularly the fields are a few meters from houses (on average ca. 10 m, Figure 3), the chance for contact between rodents (and their excreta) and humans is highly likely.

We found some indications for potential TIGV transmission between small mammals and humans, as six out of the 114 tested persons (5.26%) carried PUUV reactive antibodies in their blood. The seroprevalence in this sampled population was in the same range as other newly discovered orthohantaviruses in Africa [10]. Of note, from the serological analysis, it was not possible to be certain whether the detected antibodies were generated in response to TIGV or a different unidentified virus; our results have to be confirmed by using other immune-based assays or molecular characterization [10,12]. The white-footed Ethiopian rat is the main reservoir of the TIGV, and is common and often found in and around human inhabitations in the study areas [7,22]. Additionally, since all the seropositive people had lived their whole life in the same area where the TIGV had been discovered and most of them never travelled for long periods outside their villages, they had likely been infected within the study areas. Taken together, our results therefore suggest that the detected antibodies were directed against TIGV.

Five out of the six human seropositive samples came from Atsbi, with a seroprevalence of 10.20% compared to 1.54% in Hagere Selam. Nevertheless, all positively tested rats were found in Hagere Selam. The two study areas also differed in stone bund density and length (Table 5 and Table 6). The small sample size, in combination with the low seroprevalence, yielded low statistical power for establishing associations between seroprevalence and potential risk factors. However, the direction of several of the associations was as expected and worth noting and investigating in further studies.

When considering those potential risk factors that did not differ significantly between the two study areas, women had higher odds of being seropositive than men, possibly due to differences in living habits. Women in the study areas spend more of their time doing household activities in or around the households compared to men. It is in the villages and households that people might come in contact with rodents and their excretions, and stored grain, garden and other resources attract rodents into households [37]. Most participants and all the seropositive participants did see rodents in or around their house or grain storage areas.

Although rodents were common around houses, most participants have never had contact with rodents (72/114) and had never been bitten by rodents (104/113). Nevertheless, even though not significant, the odds of being seropositive were highest (OR: 2.83) among those who have had contact with rodents once per year or more often than those who have had contact with rodents once per year or never (OR: 0.5–1.0), and among those who had been bitten by rodents (OR: 2.48) than those who had never been bitten by rodents (OR: 1.0). In Atsbi, where cats were common, the risk for being seropositive was by about fivefold higher among those who did not have a cat compared to those with cats. These results suggest that risk factors for orthohantaviruses are related to exposure to rodents [21].

When considering the stone bunds in the Atsbi area where most seropositive humans were living, the results showed that SBD was on average lower among the seropositives than among the seronegatives (Table 5). This result was unexpected considering that rodent abundance is positively associated with SBD [27]. Furthermore, those who lived in houses where the stone bunds were reported to be 10 years or younger also had higher odds of being seropositive (Table 4). One potential explanation for this unexpected result might be that despite having a locally lower density in the LSBD areas than in the HSBD areas, rodents from the LSBD areas are more prone to infesting human inhabitations during periods of no crop cover in the fields than the rodents in the HSBD, because there is less shelter (and food) in the areas with LSBD. The chance of being bitten by rodents was slightly higher among individuals residing in the houses with stone bunds < 10 years than those residing in houses with stone bunds > 10 years.

## 5. Conclusions

This study was the first attempt to examine orthohantavirus-reactive antibodies in humans and small mammals in Ethiopia and, despite the limited sample size, we report, for the first time, the detection of orthohantavirus-reactive IgG antibodies in six humans and two rats in the country. The next step would be to screen for the orthohantavirus RNA and antibodies against orthohantaviruses among persons and small mammals from the two study areas using a larger sample size. At a local scale, stone bunds seem to be involved in determining the risk of being infected by the orthohantavirus, but not in the way that was hypothesized based on previous studies. The results suggest that stone bund density is negatively associated with seroprevalence. This study upholds the previous report that the white-footed Ethiopian rat is likely the sole host of TIGV RNA at the elevations studied, as the other co-occurring small mammals have all tested negative for the virus. Large-scale studies, on both humans and the small mammals, would be valuable regardless of whether the orthohantavirus turns out to cause a disease or not. If it does, knowledge about its temporal and spatial distribution and the risk factors for infection is crucial to be able to design and adapt preventive measures. If not, it could be used as a safe model system to study transmission and epidemiology of orthohantaviruses in general. We suggest that rural extension services creating awareness among farmers about rodent pest management practices should be encouraged to demonstrate not only the impacts of rodents as crop pests but also as hosts of rodent-borne agents potentially pathogenic to humans.

## Figures and Tables

**Table 1:** Variables used in the statistical analysis.

Variable	Type of Data Collected	Categories	Definition/Explanation
Serostatus (dependent)	Categorical, via IFA analyses of blood samples	Positive Negative	Samples containing orthohantavirus-reactive antibodies were considered to be positive
Study area	Categorical, via Questionnaire	Atsbi Hagere Selam	The two areas where TIGV was first discovered in rodents
Gender	Categorical, via Questionnaire	Women Men	
Age	Quantitative, discrete, via questionnaire categorized in statistical analyses	18–40 years 41–60 years 61–80 years	
Stone bund age	Categorical, via questionnaire	≤10 years >10 years	Age of majority of stone bunds that belonged to the household. Data collected per household
Stone bund density (SBD)	Quantitative, continuous. Assessed via GPS point and GIS.	-	Length (m) of stone bunds within 100 m from human dwellings. Data assessed per household
Cat	Categorical, via questionnaire	Yes No	Presence of domestic cat(s) in the household
Sightings of rodents	Categorical, via questionnaire	In house or storage Never in house or storage	Location of where participants did see rodents
Contact with rodents	Categorical, via questionnaire	Never touch Touch < once/year Touch ≥ once/year	Whether participants ever touched rodents, dead or alive, and in such case how often
Rodent bite	Categorical, via questionnaire	Yes No	Whether participants had ever been bitten by a rodent

**Table 2:** Abundance (number of individual captures) of small mammals in the low stone bund density (LSBD) and high stone bund density (HSBD) fields and in the enclosures in Atsbi and Hagere Selam. Numbers in parentheses indicate relative abundances (%) of each species per study area.

Astbi					Hagere Selam			
Small Mammals Abundance								
Species	LSBD	HSBD	Enclosure	Total	LSBD	HSBD	Enclosure	Total
*Acomys cahirinus* (North East African Spiny Mouse)	-	-	-	-	-	-	6 (12.5)	6 (12.5)
*Arvicanthis niloticus* (African Grass Rat)	2 (3.57)	11 (19.64)	12 (21.43)	25 (44.64)	5 (10.42)	2 (4.17)	-	7 (14.58)
*Crocidura olivieri* (African Giant Shrew)	-	-	4 (7.14)	4 (7.14)	2 (4.17)	3 (6.25)	-	5 (10.42)
*Dendromus mystacalis* (Chestnut African Climbing Mouse)	-	-	-	-	-	-	1 (2.08)	1 (2.08)
*Mastomys awashensis* (Awash Multimammate Mouse)	-	-	-	-	3 (6.25)	1 (2.08)	-	4 (8.33)
*Mus proconodon* (Rhoads’s Pygmy Mouse)	4 (7.14)	2 (3.57)	11 (19.64)	17 (30.36)	4 (8.33)	6 (12.5)	-	10 (20.83)
*Rattus rattus* (Roof Rat)	-	-	1 (1.79)	1 (1.79)	-	1 (2.08)	-	1 (2.08)
*Stenocephalemys albipes* (White-footed Ethiopian Rat)	2 (3.57)	5 (8.93)	2 (3.57)	9 (16.07)	6 (12.5)	6 (12.5)	2 (4.17)	14 (29.16)
Total	10 (17.86)	18 (32.14)	28 (50)	56 (100)	20 (41.67)	19 (39.58)	9 (18.75)	48 (100)

**Table 3:** Number of individuals captured per species in the dry and post-rainy seasons.

Species	Atsbi			Hagere Selam			Overall
	Dry	Post-Rainy	Total	Dry	Post-Rainy	Total	
*A. cahirinus*	0	0	0	3	3	6	6
*A. niloticus*	19	6	25	7	0	7	32
*C. olivieri*	0	4	4	0	5	5	9
*D. mystacalis*	0	0	0	1	0	1	1
*M. awashensis*	0	0	0	0	4	4	4
*M. proconodon*	8	9	17	5	5	10	27
*R. rattus*	0	1	1	0	1	1	2
*S. albipes*	9	0	9	8	6	14	23
Total	36	20	56	24	24	48	104

**Table 4:** Output from bivariate logistic regression testing the association between seropositivity and the predictor variables: Study area, Gender, Age, Stone bund age, Cat, Contact with rodents and Rodent bite.

Independent Variable	Characteristics	Sample Size (% of Study Population)	Seropositive No. (%)	Logistic Regression					
				Total Study Population			Only Atsbi Population *		
				Odds Ratio (OR)	95% CI of OR	*p*-Value	Odds Ratio (OR)	95% CI of OR	*p*-Value
Study population	Overall	114 (100)	6 (5.26)	7.27	0.82–64.4	0.075			
Study area	Atsbi	49 (43)	5 (10.20)	7.27	0.82–64.4	0.075			
	Hagere selam	65 (57)	1 (1.54)	1	-	-			
Gender	Women	78 (68)	5 (6.4)	2.40	0.27–12.3	0.443	1.9	0.19–18.3	0.592
	Men	36 (32)	1 (2.3)	1	-	-	1	-	-
Age	18–40	57 (50)	2 (4.0)	1	-	-	1	-	-
	41–60	35 (31)	2 (5.7)	1.67	0.22–12.4	0.618	0.5	0.04–6.02	0.585
	61–80	22 (19)	2 (9.1)	2.75	0.36–20.8	0.328	2.25	0.27–18.9	0.455
Stone bund age (*n =* 108 **)	≤10 year	37 (34)	4 (10.8)	8.48	0.91–79.9	0.060	5.25	0.50–54.8	0.166
	>10 year	71 (66)	1 (1.4)	1	-	-	1	-	-
Cat	Yes	69 (61)	4 (5.7)	1	-	-	1	-	-
	No	45 (39)	2 (4.4)	0.76	0.13–4.31	0.753	5.25	0.39–71.4	0.213
Contact with rodents	Never touch	72 (63)	4 (5.6)	1	-	-	1	-	-
	Touch < once/year	35 (31)	1 (2.3)	0.50	0.05–4.65	0.542	0.42	0.04–4.38	0.469
	Touch ≥ once/year	7 (6)	1 (14.3)	2.83	0.27–29.5	0.384	8.00	0.39–164	0.177
Rodent bite (*n =* 113 ***)	Yes	9 (8)	1 (11.1)	2.48	0.26–23.8	0.443	1.32	0.13 –13.7	0.815
	No	104 (92)	5 (4.8)	1	-	-	1	-	-

* Only Atsbi population is reported because Hagere Selam population had too few positive samples to perform statistics. ** Notation about Stone bund age was missing from six samples. *** Notation about Rodent bites was missing for one sample.

**Table 5:** Outputs from two sample t-tests. Comparison of average stone bund density (SBD) between Atsbi and Hagere Selam as well as comparison of average SBD between seropositive and seronegative samples.

SBD within 100 m from Houses					
	Min–Max	Atsbi	Hagere Selam	T-Test (Ho: diff = 0)	
		(±95% CI)	(±95% CI)	T	*p*-value
All samples (*n =* 113 *)	163–2033	591 (±79)	1300 (±87)	−11.7	0.000
		Seropositives	Seronegatives		
Atsbi (*n =* 49)	163–1165	442 (±199, *n =* 5)	608 (±86, *n =* 44)	1.99	0.085
Hagere Selam (*n =* 64 *)	561–2033	1504 (*n =* 1)	1297 (±88, *n =* 63)	−4.96	0.000

* Notation about Stone bund density was missing for one sample in Hagere Selam.

**Table 6:** Comparison of density and length of stone bunds for Atsbi (4.54 ha) and Hagere Selam (10.75 ha) as obtained from the drone image analysis. “-” = no such type of stone bund.

	Type	Hagare Selam		Atsbi		Density Difference	Length Difference
		Density (m/ha)	Length (m)	Density (m/ha)	Length (m)		
Outside village	Total	396.91	1803.02	437.92	4628.14	−41.01	−2825.12
In village	Total	134.08	609.08	91.17	963.48	42.91	−354.40
Overall		455.87	2070.88	461.34	4875.66	−5.47	−2804.78
Outside village	Small	176.00	799.53	63.31	669.09	112.69	130.44
	Medium	57.44	260.94	206.23	2179.50	−148.79	−1918.56
	Big	163.46	742.55	168.38	1779.54	−4.92	−1036.99
In village	Small	1.93	8.77	-	-	-	8.77
	Medium	-	-	8.22	86.87	-	−86.87
	Big	132.15	600.31	82.95	876.61	49.2	−276.30

**Table 7:** Output from Fisher’s exact test of the association between the variables Sightings of rodents and Serostatus, both for the total study population and only for Atsbi population.

Fishers Exact Test		Total Study Population		Only Atsbi Population *	
		Sero +	Sero -	Sero +	Sero -
Sightings of rodents	In house and storage	6	88	5	41
	Never in house or storage	0	20	0	3
	*p*-value	*p* = 0.588		*p* = 0.719	

* Only Atsbi population is reported because Hagere Selam population had too few positive samples to perform statistics.

## Data Availability

The raw data for the results presented in this study are available on request from the corresponding author.
